# Bibliometric Analysis of Global Research on Perinatal Palliative Care

**DOI:** 10.3389/fped.2021.827507

**Published:** 2022-01-20

**Authors:** Yiting Wang, Chunjian Shan, Yingying Tian, Congshan Pu, Zhu Zhu

**Affiliations:** ^1^Department of Obstetrics, Women's Hospital of Nanjing Medical University, Nanjing Maternity and Child Health Care Hospital, Nanjing, China; ^2^Department of Neonatology, Women's Hospital of Nanjing Medical University, Nanjing Maternity and Child Health Care Hospital, Nanjing, China; ^3^Department of Nursing, Women's Hospital of Nanjing Medical University, Nanjing Maternity and Child Health Care Hospital, Nanjing, China

**Keywords:** perinatal palliative care, perinatal hospice, bibliometric, web of science, VOSviewer

## Abstract

**Objectives:**

The aim of this study was to perform a bibliometric analysis of publications related to perinatal palliative care to identify scientific output and research trends at a global level.

**Methods:**

The Web of Science Core Collection database was searched to retrieve publications focusing on perinatal palliative care published between 2001 and 2020. All retrieved publications were identified by title and abstract for their relevance to perinatal palliative care. These eligible publications were extracted from the following data: title, abstract, year, keywords, author, organization, journal and cited literature. VOSviewer software was used to conduct bibliographic coupling, coauthorship, and cooccurrence analyses and to detect publication trends in perinatal palliative care research.

**Results:**

A total of 114 publications concerning perinatal palliative care were included. The annual number of publications has increased dramatically in recent years. The United States has made the largest contribution to this field with the majority of publications (68, 59.6%) and citations (1,091, 70.5%) and with close collaborations with researchers in Canada, Portugal and Australia. Wool C and her institution, York College of Pennsylvania, are the respectively, most prolific author and institution in this field, publishing 18 papers (15.8%). Journal of Palliative Medicine is the leading and main journal in this field. According to the cooccurrence network analysis, five main research topics were identified: the candidates for PPC, service models and forms, framework components, parental perspectives and satisfaction, and challenges and needs of health care providers.

**Conclusion:**

The findings of this bibliometric study illustrate the current state and global trends of perinatal palliative care for the past two decades, which will help researchers determine areas of research focus and explore new directions for future research in this field.

## Introduction

According to data released by the World Health Organization (WHO), an estimated 2.4 million infants died during the neonatal period in 2019, with the neonatal mortality rate reaching 17 per 1,000 live births (1). Notably, congenital malformations and chromosomal abnormalities were the leading causes of infant death, accounting for 20.8% of all infant deaths (2). With advances in prenatal screening and diagnostic techniques, these congenital abnormalities can be detected early, making more parents aware during pregnancy that their child is likely die at birth or shortly after (3). Despite receiving a prenatal diagnosis of a life-limiting condition (LLC), many families choose to continue their pregnancy based on their own beliefs, values and expectations. Caring for these infants and families poses a major challenge to health providers in obstetric and pediatric settings. This has prompted the development of perinatal palliative care (PPC) as an emerging field of palliative care to address the previously unmet needs of these families.

Perinatal palliative care refers to a coordinated and interdisciplinary care strategy dedicated to maximizing the quality and comfort of life of fetuses diagnosed prenatally as life-limiting and providing supportive care to their families throughout the pregnancy, delivery and postnatal periods (4). The World Health Assembly decided that providing palliative care for children is “an ethical responsibility of health systems” and that the integration of palliative care into health care systems can enable the achievement of the sustainable development goal of universal health coverage (5). However, there are various barriers to the development and accessibility of PPC services worldwide, including those related to public awareness, health policies, education and other conditions appropriate for PPC.

Globally, the development of PPC services varies greatly, and it is closely related to the state of PPC research in a country or region (6). Research generates the evidence necessary for the development of PPC services, whether for seeking best practices, identifying barriers and facilitators, monitoring outcomes, or determining health priorities (7). Additionally, in official documents of the WHO, research is recognized as one of the indicators used to assess and monitor the provision of PPC services (8). Hence, a better understanding of the current state of research can provide a clearer account of global trends, needs and challenges in the field of PPC.

In recent years, bibliometric analysis has been widely applied in quantitative analyses of literature research. Meanwhile, the approach can be used to describe academic activities in a research field; compare the contributions of countries, journals, institutes, and researchers; and detect future research directions (9, 10). Therefore, in this study, a bibliometric analysis of publications related to PPC was applied to identify scientific output and research trends at the global level to provide beneficial insight into policy-making and resource allocation and facilitate leading-edge, needs-led research.

## Methods

### Data Sources

Data on PPC were retrieved from the Web of Science (WoS) Core Collection (WoSCC) Database. The WoSCC is the premier resource on the WoS platform and the world's most trusted citation index for scientific and scholarly research. The resource covers a collection of more than 21,000 peer-reviewed, high-quality scholarly journals, including open access journals, published worldwide in over 250 sciences, social sciences and humanities disciplines. Conference materials and book data are also available. The WoSCC was chosen for the present search, with all authors and their affiliations, abstracts and keywords, funding acknowledgments, cited references and unified institution names were captured for each paper, allowing for an in-depth exploration of specialized scholarly fields.

### Search Strategy

For the search, the terms (perinatal^*^ OR antenatal^*^ OR prenatal^*^ OR fetal^*^ OR fetal^*^ OR perinatolog^*^) AND (palliative care OR hospice^*^) were used as “topics” (titles, abstracts, author keywords, and keywords plus). The search time span was set to January 2000 to December 31, 2020, and editorial materials, letters, and meeting abstracts were excluded. Due to a considerable number of publications written in French, no language limits were applied when searching.

### Data Collection

**Figure 1:**
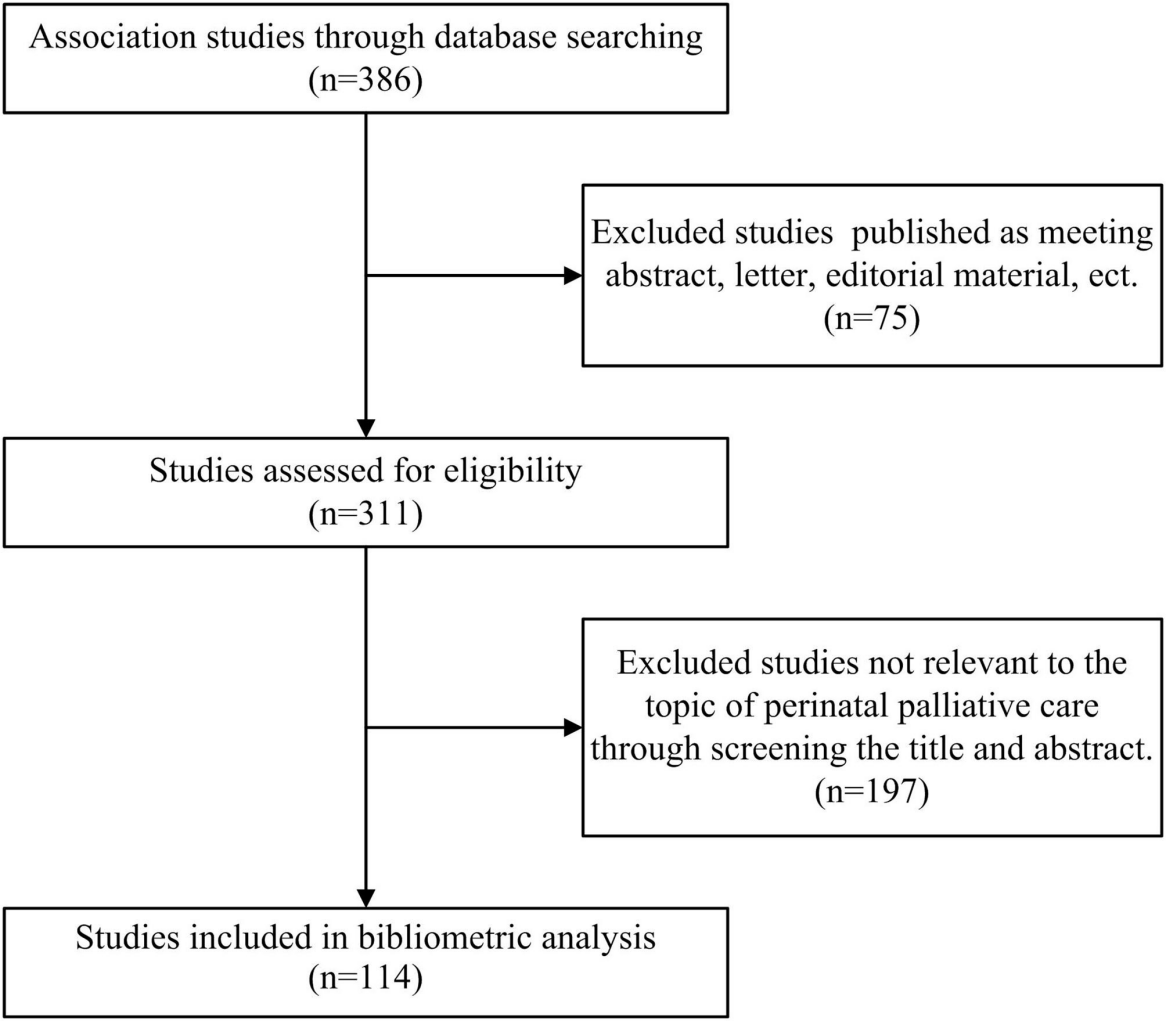
Flow diagram of literature search.

Publications were first screened by title and abstract for their relevance to PPC, which provides palliative comfort care from the prenatal diagnosis of a life-limiting fetal condition. Studies exclusively addressing neonatal palliative care of extremely premature cases and pediatric palliative care were excluded. The article screening process was performed independently by two researchers (C.P. and Y.W.), and any disagreement was resolved in a consensus meeting with held Y.T. After the eligible articles were identified, the “full records and cited references” of the articles were exported from the WoSCC on April 30, 2021. The process of articles selection used is shown in Figure 1. The following information was recorded: title, authors, year of publication, journal, country of origin, affiliation, keywords, total number of article citations, and research field.

### Bibliometric and Visualized Analysis

This study applied VOSviewer (version 1.6.16), which has been widely used in recent bibliometric studies (11–14), to construct and visualize bibliometric networks. VOSviewer is a freely available software tool for creating maps based on network data and for visualizing and exploring these maps. In the generated visualization map, different colors reflect different clusters. The size of a label and the circle for an item are determined by an item's weight. The distance between two items reflects the strength of the relation between the items. Connecting lines represent parameters such as cooperation and cocitation. The stronger the link between two items is, the thicker the line used to display the link is, providing easily interpretable graphical representations (15).

### Ethics

Ethics review board's approval was exempted because the study involved collecting and analyzing data from a public network platform and did not involve human subjects.

## Results

A total of 114 articles concerned with perinatal palliative care published between 2001 and 2020 were retrieved from the WoSCC database. Articles (78, 68.4%) were the most common publications identified, followed by reviews (31, 27.2%), case reports (4, 3.5%), and guidelines (1, 0.9%).

### Annual Growth Trend of Publications

**Figure 2:**
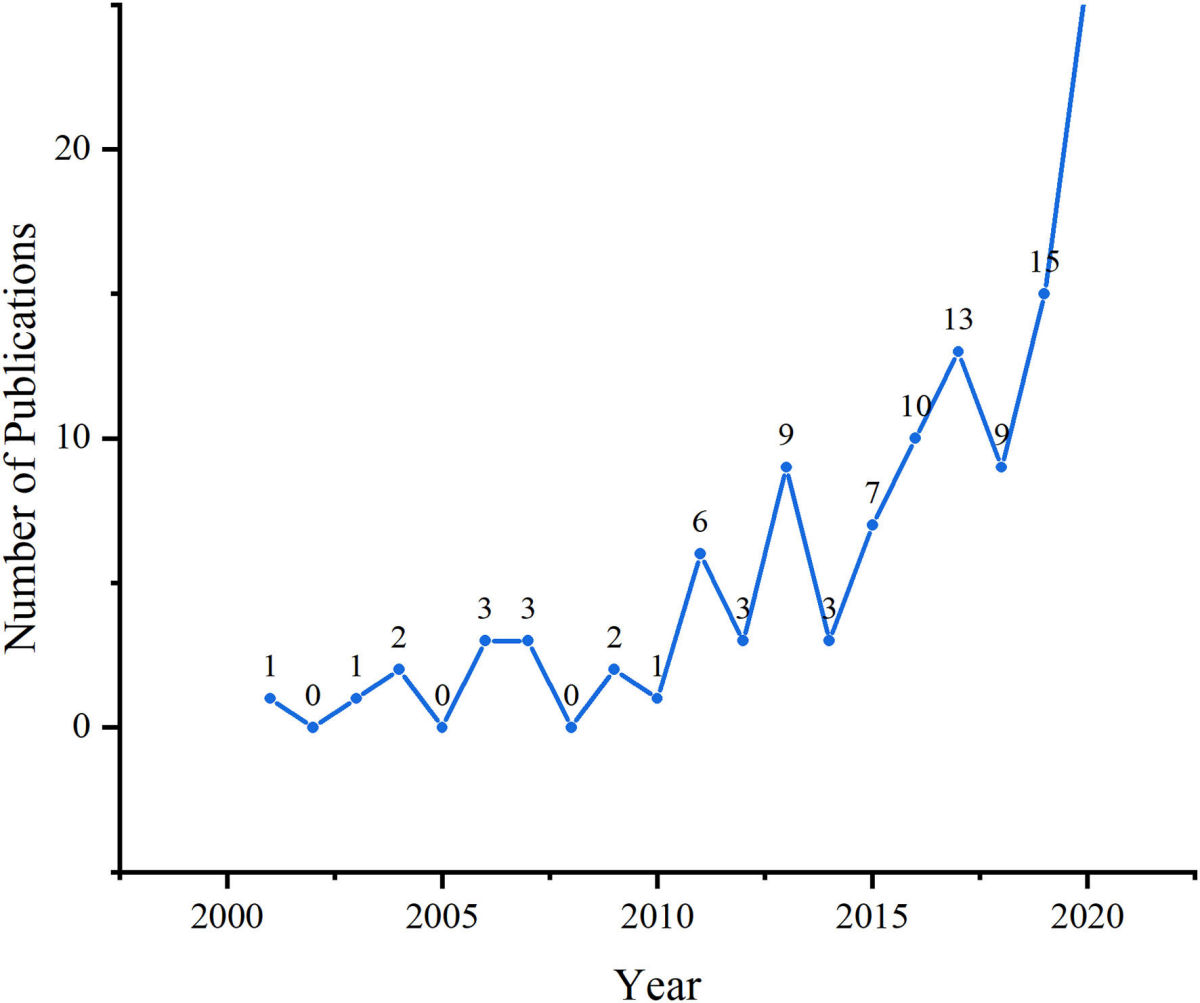
Growth trend of annual publications from 2001 to 2020.

Figure 2 shows the growth trend of annual publications related to perinatal palliative care research for 2001–2020. The average number of publications was recorded as 5.7 documents per year. In the decade of 2001–2010, there were only a few publications, averaging 1.3 documents per year, for a total of 13 documents. From 2011 to 2015, the volume of publication fluctuated but generally increased. A total of 28 publications were published in the 5-year period, which is more than twice that of the previous decade. Except in 2018, annual publications increased steadily from 2016 to 2020. Moreover, more than half of the articles were published after 2016, indicating drastic growth in publications. Productivity peaked in 2020 with 26 publications (22.8% of the total).

### Geographical Distribution of Publications

Table 1 shows countries of origin (17 countries) for the 114 publications. Worldwide, perinatal palliative care has been studied in only a few countries, and most of them are high-income countries. The United States contributed the most articles (68 articles, 59.6% of the total), followed by France (11 articles) and Canada (nine articles). The top three countries contributed 77.2% of all publications. Research on perinatal palliative care in the United States, France, Canada and the United Kingdom developed early, with first publications published before 2010. Among these countries, the United States and Canada were involved in more frequent international research collaborations with other countries. The two countries also produced the most citations. The United States and Canada are the two countries the most actively involved in international cooperation. The United States most frequently collaborates with Canada (3), Portugal (2), and Australia (1). In addition to collaborating with the United States, Canada has been involved international cooperation with France (1) and Ireland (1). Switzerland only has intensive cooperation with Germany (2).

**Table 1:** Geographical distribution of publications and international research collaborations in 17 countries/regions.

**Country**	**Documents (%)**	**Year of first publication**	**Citations**	International collaborations^*^
United States	68 (59.6)	2001	1091	6
France	11 (9.6)	2009	66	1
Canada	9 (7.9)	2009	164	5
United Kingdom	5 (4.4)	2007	77	0
Switzerland	4 (3.5)	2016	12	2
Ireland	4 (3.5)	2018	11	1
Australia	3 (2.6)	2011	14	2
Poland	3 (2.6)	2013	7	0
Germany	3 (2.6)	2018	4	2
Italy	3 (2.6)	2020	0	0
Portugal	2 (1.8)	2017	12	3
Brazil	2 (1.8)	2017	12	0
Spain	1 (0.9)	2012	75	0
Czech Republic	1 (0.9)	2019	2	0
Sweden	1 (0.9)	2020	1	0
Hungary	1 (0.9)	2020	0	0
Iran	1 (0.9)	2020	0	0

* *Accumulated number of international collaborations*.

### Institutions

The institutions that have most contributed to PPC research globally are presented in Table 2. The York College of Pennsylvania was the most productive institute, with 18 documents (15.8%), followed by the University of North Carolina (including the Chapel Hill and Greensboro campuses) and Aix-Marseille University (*n* = 6, 5.3%). Furthermore, the York College of Pennsylvania was found to be the most critical institution in the cooperation network and enjoys a high level of citations. However, on the basis of the funding rates received by all publications, two of the top three most productive institutions had the lowest funding rates of 16.7 and 0.0%.

**Table 2:** Most productive institutions in the field of PPC research.

**Institutions**	**Countries**	**Publications**	**Funded (%)**	**Citations**	Collaborations^*^
York Coll Penn	USA	18	3 (16.7)	209	26
Univ N Carolina	USA	6	4 (66.7)	84	16
Aix Marseille Univ	France	6	0 (0.0)	45	13
Univ Rochester	USA	5	4 (80.0)	137	14
Univ Montreal	Canada	5	2(40.0)	132	10
Children's Hosp Wisconsin	USA	4	1 (25.0)	143	7
Univ Illinois	USA	4	3 (75.0)	88	15
Children's Hosp Philadelphia	USA	4	1 (25.0)	81	9
Univ Calif San Diego	USA	4	1 (25.0)	52	4

* *Accumulated number of interinstitution collaborations*.

### Authors

In this study, all authors were included in the analysis without considering authors' relative contributions to a single study. Each author's full name was extracted to minimize errors. The author Wool C was the most active among the authors, contributing to 18 publications. Both Cote-Arsenault D and Tosello B ranked second, with six publications (Table 3). Additionally, Wool C was the most cited author, with 214 citations, followed by Cote-Arsenault D and Leuthner SR. The h-index is widely used to measure both the productivity and citation impact of publications. In the current study, the h-index for Wool C was recorded as eight in the PPC field. That is, each of the author's eight publications has received at least eight citations. For the coauthorship analysis, the results show that Wool C had the largest research team with 21 coauthors. These indicators again confirm the academic influence of Wool C.

**Table 3:** Authors publishing more than 2 articles on perinatal palliative care.

**Author**	**Countries**	**Publications**	**Citations**	H-index^*^	**Coauthors**
Wool, Charlotte	USA	18	214	8	21
Cote-Arsenault, Denise	USA	6	141	4	9
Tosello, Barthelemy	France	6	51	4	12
Leuthner, Steven R.	USA	5	184	4	3
Denney-Koelsch, Erin M.	USA	5	137	4	6
Kavanaugh, Karen	USA	5	92	4	11
Marc-Aurele, Krishelle Leong	USA	4	52	3	6
Einaudi, Marie-Ange	France	4	47	4	11
Gire, Catherine	France	4	47	4	11
Betremieux, Pierre	France	4	45	4	19
Carter, Brian S.	USA	4	22	2	8
Farlow, Barbara	Canada	3	120	3	7
Janvier, Annie	Canada	3	120	3	7
Black, Beth Perry	USA	3	48	3	6
Kuebelbeck, Amy	USA	3	4	1	6

* *According to the Web of Science Core Collection Database*.

### Journals

The 114 articles were published in 56 journals, with 10 published in the Journal of Palliative Medicine (8.8%), eight published in Frontiers in Pediatrics (7.0%) and seven published in JOGNN-Journal of Obstetric Gynecologic and Neonatal Nursing (6.1%). Table 4 lists journals publishing more than two articles. Of these journals, the Journal of Palliative Medicine has the most citations at 182, and Archives of Disease in Childhood-Fetal and Neonatal Edition presents the highest impact factor of 5.747.

**Table 4:** Journals publishing more than 2 articles on perinatal palliative care.

**Journal**	**Documents**	**Citations**	IF-2020^*^
Journal of Palliative Medicine	10	182	2.947
Frontiers in Pediatrics	8	0	3.418
JOGNN-Journal of Obstetric Gynecologic and Neonatal Nursing	7	124	1.716
Journal of Perinatal & Neonatal Nursing	6	133	1.638
Advances in Neonatal Care	5	20	1.968
Archives de pediatrie	5	15	1.180
Perspectives in Biology and Medicine	5	0	1.416
Journal of Maternal-Fetal & Neonatal Medicine	4	23	2.398
Archives of Disease in Childhood-fetal and neonatal edition	3	81	5.747
MCN-The American Journal of Maternal-Child Nursing	3	70	1.412
Seminars in Perinatology	3	37	3.300
Midwifery	3	12	2.372

* *IF denotes the impact factor from Thomson Reuters' Journal Citation Reports (JCR)*.

### Keywords

**Figure 3:**
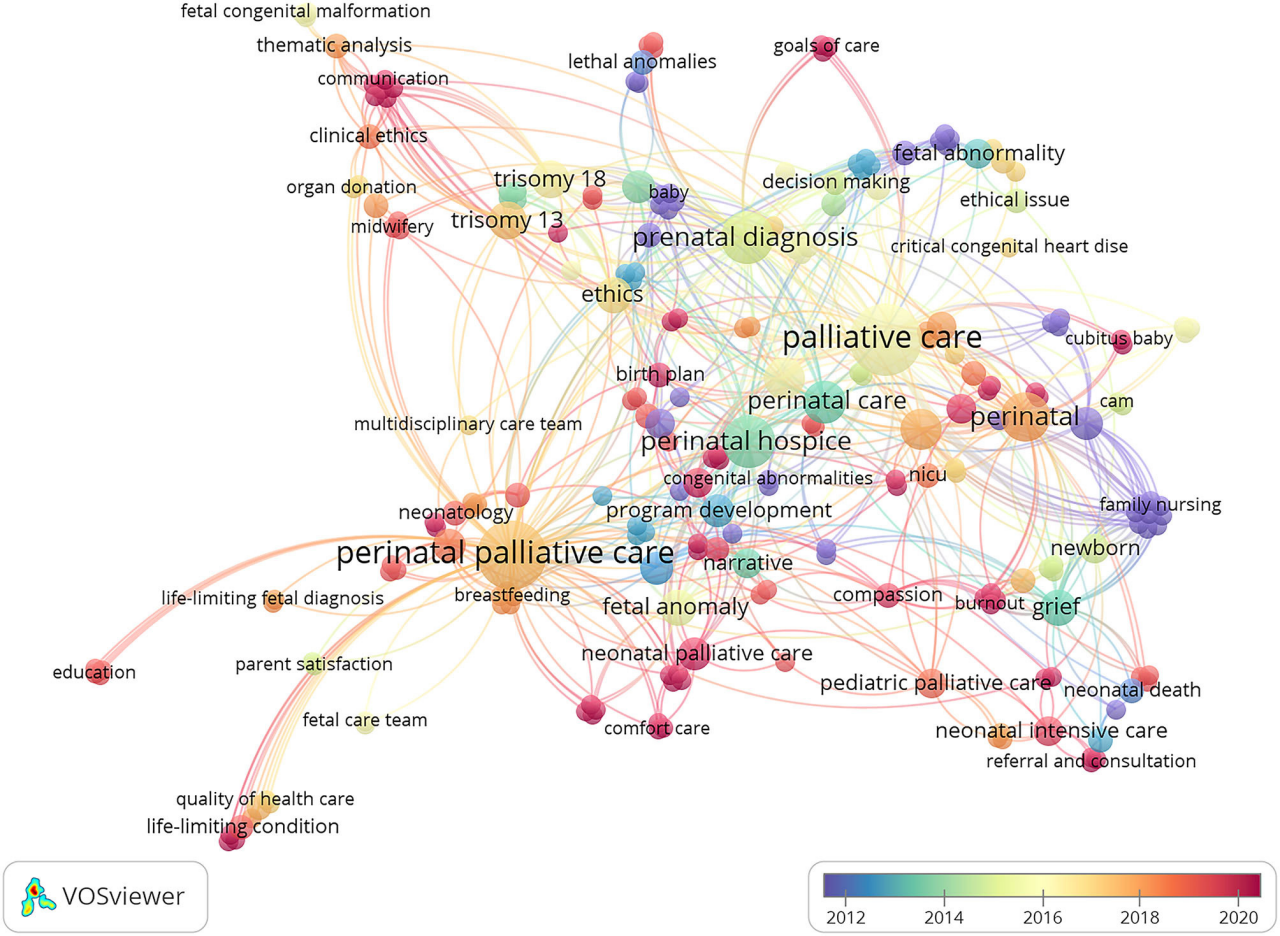
Cooccurrence network of author keywords in global PPC research.

Of the 114 included studies, 34 did not include author keywords, so only 80 were included in the keyword analysis. As shown in Figure 3, labels and circles for palliative care, perinatal palliative care and perinatal hospice are the largest in the cooccurrence network of author keywords, showing the three keywords to be the most frequently used keywords, occurring more than 13 times. In addition, some topics of research can be identified, including (i) candidates for PPC; (ii) PPC models and forms; (iii) components related to the PPC framework, including prenatal counseling, decision-making, birth planning and bereavement; (iv) parental perspectives and satisfaction; and (v) challenges and needs of health care providers. All of these topics have been extensively studied in this domain. In analyzing keyword trends, VOSviewer was used to create a visual map of the cooccurrence network, which uses colors to mark keywords based on their average periods of appearance in all included publications. Blue color means the keywords appear earlier, while red color means the keywords appear later. Red keywords also reflect the latest progress in PPC research (e.g., community palliative care, communication, goals of care, birth planning, compassion, etc.).

## Discussion

This study conducted a comprehensive analysis of perinatal palliative care, not only providing a historical overview of scientific progress but also revealing research trends (16). In view of the observed growth in publications, the field of PPC is still quite new, with literature starting to appear in the 2000's and with the concept of perinatal hospice first proposed in 1997 (17). The number of publications has since grown slowly over time. It was not until 2010 that the literature in the field grew dramatically, which seems to be due to the development of palliative care research worldwide. With the release of the PPC guidelines and the growing maturity of service models, it is reasonable to expect the growth of the literature to continue (4).

In terms of the geographic distribution of publications, North America and Europe are the regions where PPC studies were published earlier and have been published more frequently. Within these regions, the United States plays a leading role, and France and Canada have also made significant contributions. In South America, Oceania, and Asia, only Brazil, Australia and Iran have been leaders in conducting PPC research. Africa has made almost no contributions. This reveals that international research on PPC is mainly concentrated in high-income countries, while relevant research activity and resources in middle- and low-income countries are still scarce. To considered different behaviors and cultures, it would be beneficial to extend collaboration networks to more geographic regions (18). Meanwhile, all countries must be encouraged and empowered to develop their own models of perinatal palliative care delivery based on their unique resources and conditions rather than advocating a one-size-fits-all model of PPC (19).

International cooperation and collaboration are the general trend and mainstream direction of the development of science and technology, which can promote the global sharing of information and resources and accelerate the development of academic fields. However, this study shows that international collaboration has not been as widespread as expected, with only 8 (7.0%) of the identified publications resulting from international collaboration. The United States and Canada are highly active in international collaboration and not only cooperate closely with each other but also with other geographically proximal countries, such as Ireland, France and Portugal. Similar to what is found in other fields, research collaboration between countries shows obvious geographic clustering characteristics (20). The reason may be that geographic proximity and cultural similarity may provide a good basis for international cooperation and collaboration. We look forward to more frequent research collaborations between different countries/regions in subsequent studies to obtain evidence based on national and even international clinical studies to benefit more families facing fetal life-limiting diagnoses.

The leading 10 research institutions are from the leading three countries in terms of publication numbers. The same phenomenon was found for authors with almost all researchers from leading institutions. These results indicate that leading institutions and researchers have played an important role in improving academic levels in their respective countries. Furthermore, most of the identified institutions and researchers are based in the USA, which again shows the academic influence of the USA in this field. However, we found that the discipline has struggled to obtain sufficient support and funding, which may be related to poor understanding of PPC among the public, professionals and policy-makers (21). Most top institutions, especially Aix-Marseille University, the top French institution in this field, have not received funding for all relevant publications. Even for the York College of Pennsylvania, which has produced the most articles, only 16.7% of the identified studies were funded. Therefore, earmarked funds should be focused in this specialized area to allow more institutions and researchers to develop robust evidence and foster diversity in this field. On the other hand, with ongoing research, it is difficult for a single person or institution to achieve major breakthroughs, and there is an urgent need for researchers and institutions in this field to cooperate with each other (22). Therefore, many associations have provided financial support to scholars worldwide through the Traveling Scholarship Program and Traveling Fellowship Program to strengthen palliative care messaging, information and communication to inspire global change (19).

Almost all PPC-related studies have been published in specialized journals focused on obstetrics and gynecology, perinatology, neonatology/pediatrics, palliative medicine and nursing. This reflects the increasingly multidisciplinary nature of PPC. The number of articles is generally lower than for other specialties of perinatology and palliative medicine. Furthermore, most articles have been published in journals of relatively low impact relative to those of palliative care in other fields, such as cancer (23), surgery (24), and geriatric care (25), revealing a lack of global attention to this topic.

All author keywords included in our study were analyzed to generate a cooccurrence network map. With this network map, there is an opportunity to further clarify future development trends. Historically, many articles initially concentrated on candidates for perinatal palliative care. Thus, “lethal anomalies,” “congenital abnormalities” and “life-limiting fetal diagnoses” were the most commonly used keywords, and some articles even extended to certain diseases, such as trisomy 13, trisomy 18 and anencephaly (26, 27). While there is a general consensus on lethal prenatal diagnoses, with advances in fetal and neonatal medicine, we must be wary of referring to some diseases as “lethal.” Future research on the treatment and prognosis of certain “lethal” diseases may provide more information that can help families make decisions. Additionally, the present study found that researchers have conducted many studies on the development of perinatal palliative care service models. As the most frequently used keywords, family-centered care and interdisciplinary care have a strong link to program development. Perinatal palliative care takes many forms, such as antenatal palliative care, neonatal palliative care and pediatric palliative care, and it varies by the location of service. Recently, community palliative care has been considered a “new frontier” that supports families longitudinally and across various care settings (3, 28). As indicated in previous studies, community hospitals have significant advantages in providing more care and more accessible options for families with short infant life expectancy ^19^. Moreover, a variety of components related to the PPC framework, including prenatal counseling, decision-making, birth planning and bereavement, have dominated the field of research. In the past, publications related to decision-making and bereavement were popular, but in recent years, publications on birth planning have become more prominent. According to the current study, many areas of birth planning still need to be explored, such as communication, follow-through and the flexibility of important components (29). In PPC settings, the measurement of nursing outcomes brings new challenges. The evaluation of PPC relies on parent-reported outcomes rather than on “hard” epidemiologic data (30), and most of the evidence comes from qualitative studies. In providing measurable indicators, the instrument development of parental satisfaction and quality indicators has also gained considerable attention in the last 10 years (30, 31). These instruments, however, still need to be validated and applied to a broader and diverse sample to improve the quality of care across settings. Despite the importance of PPC, advocating for it around the world is a very challenging proposition. From the obtained keyword network, terms such as confidence, compassion, burnout, and education highlight the challenges and needs of health care providers. Although some of these dilemmas may be difficult to overcome in the short term, significant measures must be implemented in the future to enhance providers' confidence and competence in providing good PPC (32, 33).

## Strengths and Limitations

This is the first study to attempt to apply VOSviewer software to conduct a bibliometric analysis and provide a visual account of global PPC services. However, this study presents limitations. Due to the limitations of the employed software, this study only involved research and analysis through the WoSCC database. Therefore, the data used may not be comprehensive. In addition, in the keyword analysis, some studies with no author keywords were not included in the cooccurrence analysis, which may affect the accuracy of the research results.

## Conclusion

The current study has summarized the current research status and global trends of the field of PPC research. According to the presented findings, the number of PPC studies has been increasing in the past two decades, but such research is mainly concentrated in high-income countries, while few studies have been conducted in low- and middle-income countries. To promote the development of PPC services worldwide, much work still needs to be done by strengthening international and regional research cooperation; increasing financial support; and carrying out leading-edge, demand-oriented research.

## Author Contributions

ZZ designed the study. YW and CP executed literature retrieval and screening. CS and YT conducted the statistical analysis. YW wrote the original draft of the paper, which was revised by CS and ZZ. All authors have approved of the final version of the draft.

## Funding

This work was partly supported by the Medical Science and Technology Development Foundation of Nanjing Department of Health (YKK20136).

## Conflict of Interest

The authors declare that the research was conducted in the absence of any commercial or financial relationships that could be construed as a potential conflict of interest.

## Publisher's Note

All claims expressed in this article are solely those of the authors and do not necessarily represent those of their affiliated organizations, or those of the publisher, the editors and the reviewers. Any product that may be evaluated in this article, or claim that may be made by its manufacturer, is not guaranteed or endorsed by the publisher.
